# Recombinant Production of *Pseudomonas aeruginosa* Rhamnolipids in *P. putida* KT2440 on *Acetobacterium woodii* Cultures Grown Chemo-Autotrophically with Carbon Dioxide and Hydrogen

**DOI:** 10.3390/microorganisms12030529

**Published:** 2024-03-06

**Authors:** Jonas Widberger, Andreas Wittgens, Sebastian Klaunig, Markus Krämer, Ann-Kathrin Kissmann, Franziska Höfele, Tina Baur, Tanja Weil, Marius Henkel, Rudolf Hausmann, Frank R. Bengelsdorf, Bernhard J. Eikmanns, Peter Dürre, Frank Rosenau

**Affiliations:** 1 Institute of Pharmaceutical Biotechnology, Ulm University, Albert-Einstein-Allee 11, 89081 Ulm, Germany; jonaswwidberger@aol.de (J.W.); markus-1.kraemer@uni-ulm.de (M.K.); ann-kathrin.kissmann@uni-ulm.de (A.-K.K.); 2 Max-Planck-Institute for Polymer Research Mainz, Ackermannweg 10, 55128 Mainz, Germany; weil@mpip-mainz.mpg.de; 3 Institute of Molecular Biology and Biotechnology of Prokaryotes, University of Ulm, Albert-Einstein-Allee 11, 89081 Ulm, Germany; franziska.hoefele@uni-ulm.de (F.H.); frank.bengelsdorf@uni-ulm.de (F.R.B.); bernhard.eikmanns@uni-ulm.de (B.J.E.); 4 Institute of Microbiology and Biotechnology, University of Ulm, Albert-Einstein-Allee 11, 89081 Ulm, Germany; tina.baur@uni-tuebingen.de (T.B.); peter.duerre@uni-ulm.de (P.D.); 5 Cellular Agriculture, Technical University Munich, Gregor-Mendel-Str. 4, 85354 Freising, Germany; marius.henkel@tum.de; 6 Fachgebiet Bioverfahrenstechnik (150k), Institut für Lebensmittelwissenschaft und Biotechnologie, University Hohenheim, Frühwirthstraße 12, 70599 Stuttgart, Germany; rudolf.hausmann@uni-hohenheim.de

**Keywords:** rhamnolipid, *Pseudomonas putida*, biosurfactants, anaerobic gas fermentation, *Acetobacterium woodii*, sequential fermentation, heterologous production

## Abstract

The establishment of sustainable processes for the production of commodity chemicals is one of today’s central challenges for biotechnological industries. The chemo-autotrophic fixation of CO_2_ and the subsequent production of acetate by acetogenic bacteria via anaerobic gas fermentation represents a promising platform for the ecologically sustainable production of high-value biocommodities via sequential fermentation processes. In this study, the applicability of acetate-containing cell-free spent medium of the gas-fermenting acetogenic bacterium *A. woodii* WP1 as the feeder strain for growth and the recombinant production of *P. aeruginosa* PAO1 mono-rhamnolipids in the well-established nonpathogenic producer strain *P. putida* KT2440 were investigated. Additionally, the potential possibility of a simplified production process without the necessary separation of feeder strain cells was elucidated via the cultivation of *P. putida* in cell-containing *A. woodii* culture broth. For these cultures, the content of both strains was investigated by examining the relative quantification of strain-exclusive genes via qPCR. The recombinant production of mono-rhamnolipids was successfully achieved with maximum titers of approximately 360–400 mg/L for both cell-free and cell-containing *A. woodii* spent medium. The reported processes therefore represent a successful proof of principle for gas fermentation-derived acetate as a potential sustainable carbon source for future recombinant rhamnolipid production processes by *P. putida* KT2440.

## 1. Introduction

For almost every sector of modern industry, surfactants have become an indispensable component, which is evidenced by a worldwide production in the order of 18 million tons per year and the wide range of applications they are used in, including the food industry, agriculture, medicine, and healthcare, as well as textiles and bioremediation [1,2,3,4,5]. However, as most commercial surfactants are still produced from nonrenewable petrochemical precursors, a major focus in recent decades has been on the discovery and commercialization of biosurfactants, which are almost exclusively of microbiological origin and produced from renewable substrates [6]. Among the currently available classes of biosurfactants, rhamnolipids are of particularly high industrial potential [7]. Rhamnolipids show potential for many possible applications, as reviewed extensively by Lang and Wullbrandt [8] as well as Maier and Soberón Chávez and others [9,10]. A prominent example is the use of rhamnolipids as detergents in washing agents and the cleaning sector due to their potent tensidic properties [11]. Also possible is the utilization as emulsifiers in the cosmetic and food industry [12,13]. In comparison to conventional surfactants obtained through chemical synthesis, rhamnolipids show lower toxicity, are biodegradable, and possess novel properties, such as antimicrobial effects [14,15].

The molecular structure of rhamnolipids is composed of either one (mono-rhamnolipids) or two (di-rhamnolipids) L-rhamnose sugar residues linked to one or, more commonly, two 3-hydroxyfatty acids via a β-glycosidic bond, providing them with basic structural diversity [16,17,18]. Additionally, different bacterial producer strains are known to produce rhamnolipids with fatty acid residues of varying characteristic lengths, typically ranging from C_8_ to C_16_ and differing in their shares of unsaturated bonds, leading to over 60 reported congeners with considerable varying physicochemical properties [19]. This rich structural and physicochemical variety offers unique potential for the production and utilization of rhamnolipid mixes with distinct physicochemical properties via selective heterologous gene expression [6].

The production of rhamnolipids was first described nearly 70 years ago for the human pathogen *Pseudomonas aeruginosa* [20,21,22,23,24,25,26], which represents the best-known native producer strain until today. Apart from *P. aeruginosa*, several bacteria from the genus *Burkholderia* are known producers of rhamnolipids too. Among them are known human pathogens, such as *Burkholderia pseudomallei* [27,28], or plant pathogens, such as *B. plantarii* [29] and *B. glumae* [30,31]. A major bottleneck for industrial-scale rhamnolipid production in these native producer strains is the complex regulation mechanism controlling rhamnolipid biosynthesis at the transcriptional level through the quorum sensing regulatory network [32,33,34] and probably other signaling mechanisms in these strains. Furthermore, the pathogenic potential of the native producer strains is another considerable disadvantage for their use as production platforms, especially if the utilization of the rhamnolipids in food and cosmetics is envisaged [18,35]. As a result, the heterologous production of rhamnolipids in nonpathogenic host organisms has been given considerable attention, with the strain *Pseudomonas putida* KT2440 being one of the most noteworthy examples [7,36]. This well-characterized strain is known for its versatile metabolism, rapid growth, and high level of solvent tolerance [37,38]. Utilizing the strains’ natural metabolic versatility, several recent studies have focused on the establishment of unusual carbon sources derived from sustainable raw materials as substrates to support the more ecological production of rhamnolipids. *P. putida* KT2440 has, for example, been successfully engineered to utilize sugars such as xylose and arabinose as part of lignocellulose hydrolysates obtained from agricultural residues [39]. In another approach, acetate found within biooil obtained from the fast pyrolysis of lignocellulosic biomass was shown to be a promising sustainable carbon source for rhamnolipid production by Arnold et al. [40].

A noteworthy source of the sustainable biological production of acetate is acetogenic bacteria, which already provide approximately 10% of the global annual acetate output [41]. Acetogens are a phylogenetically diverse group of obligate anaerobic bacteria that use the reductive acetyl-CoA or the Wood-Ljungdahl pathway as the main means for energy conservation and synthesis of cell carbon. Within this pathway, two molecules of CO_2_ are reduced to the acetyl moiety of acetyl-CoA, which is subsequently converted to acetate [42,43,44]. Apart from energy conservation realized by the production of acetate, another significant part of energy conversion for the cell is achieved via ion-gradient-driven phosphorylation coupled to the reductive acetyl-CoA pathway. Depending on the species of acetogen, either protons or sodium ions are translocated for additional ATP generation [45,46]. Various acetogens are successfully used for the production of valuable natural and recombinant products, such as the biofuels ethanol, butanol, and hexanol [47,48,49], as well as bulk chemicals aside from acetic acid, such as acetone or lactate [50,51] via the fixation of CO_2_ from gaseous substrates (CO_2_ + H_2_ or synthesis (syn) gas fermentation). Syngas rich in CO_2_ can be obtained from different renewable and sustainable sources, e.g., from the pyrolysis/gasification of organic waste substances or from the utilization of certain industrial off-gases, for example those of the steel industry [52]. Among acetogenic species, *Acetobacterium woodii* is of particular interest, as it is a well-studied model strain for the metabolism of sodium-dependent acetogenic bacteria [46,53]. Furthermore, an established set of tools is available for genetic engineering [50,51,54].

The aim of this study was to utilize the acetate produced by *A. woodii* via chemo-autotrophic gas fermentation as the carbon source for the recombinant production of rhamnolipids by *P. putida* KT2440. Cell-free *A. woodii* spent medium was used as a base for *P. putida* growth and rhamnolipid production. To realize the production of mono-rhamnolipids, the *rhlAB* genes of *P. aeruginosa* PAO1 were introduced into *P. putida* KT2440 within the plasmid vector pVLT31. The expression of *rhlAB* genes was controlled using an IPTG-inducible tac promoter. The cultivation of *P. putida* was carried out in pure *A. woodii* spent medium as well as in a 1:1 mixture of spent medium and conventional minimal medium. To investigate the potential influences of the presence of *A. woodii* cells on the growth and final rhamnolipid concentrations, the cultivation of *P. putida* was also carried out in a cell-containing spent medium. To monitor the cell population development in the cultures containing both the *A. woodii* and *P. putida* cells, the relative quantification of strain-exclusive genes was carried out during the cultivation.

## 2. Materials and Methods

### 2.1. Media and Cultivation

*Escherichia coli* DH5α, which was used for the plasmid amplification, was cultivated in Luria-Bertani (LB) medium [55] containing 10 g/L tryptone, 10 g/L NaCl, and 5 g/L yeast extract while shaking (150× *g*) or on respective agar plates at 37 °C. After autoclaving, the medium was supplemented with glucose until a final concentration of 1% *w*/*v*. For plasmid maintenance, the medium was further supplemented with 10 µg/mL tetracycline prior to inoculation. *A. woodii* was cultivated in modified DSM 135 medium [50] containing 0.20 g/L NH_4_Cl, 1.76 g/L KH_2_PO_4_, 8.44 g/L K_2_HPO_4_, 10 g/L NaHCO_3_, 3 g/L yeast extract, 0.5 g/L L-cysteine-HCl, 2 mL/L SL-9 trace element solution (Table S1) [56], 2 mL/L vitamin solution (Table S2) [57], 1 mL/L selenite-tungstate solution (Table S3) [56], and 1 mL/L resazurin (1 g/L). The preparation of the medium was conducted anaerobically via the seven-fold exchange of the atmosphere with N_2_ + CO_2_ (80:20). After autoclaving, the medium was supplemented with 0.33 g/L MgSO_4_·7H_2_O. Autotrophic cultivation was carried out in 110 mL of medium in 500-mL rubber-sealed Müller Krempel bottles. The bottles’ atmosphere was exchanged seven-fold with H_2_ + CO_2_ (67:33) to a final overpressure of 110 kPa. When the overpressure fell under 50 kPa during cultivation, the bottles were gassed again to 110 kPa. The precultures were cultivated in 5 mL of medium in rubber-sealed hungate tubes supplemented with 8 g/L fructose. The cells from these precultures were used for the inoculation of 50 mL medium in Müller Krempel bottles with the aforementioned H_2_ + CO_2_ gas atmosphere for adaptation to autotrophic growth. Cultivation in 5 mL and 50 mL tubes took place with prior supplementation of 5 µg/mL clarithromycin for plasmid maintenance. The cells from the 50-mL cultures adapted to autotrophic growth were then used as the inoculum for 110-mL main cultures. The cultivation of *A. woodii* was always conducted at 30 °C while shaking (150× *g*). For the cultivation of *P. putida* strains in fresh medium, either modified Wilms Kpi medium, modified DSM 135 medium (as described above), or a 1:1 mixture of both was utilized. In the case of cultivation in fresh media, acetate was supplemented in different concentrations from 100 to 500 mM as the carbon source. Aside from the fresh medium, the cultivation of *P. putida* strains was also carried out in cell-free or cell-containing spent medium of *A. woodii* (pMTL83251) or in a 1:1 mixture of said spent medium with fresh modified Wilms KPi medium. The cell-free medium was obtained via centrifugation (4500× *g*, RT, 30 min). The modified Wilms KPi medium [58] contained 0.5 g/L NH_4_Cl, 1.64 KH_2_PO_4_, 6.58 g/L K_2_HPO_4_, 2 g/L Na_2_SO_4_, 5 g/L (NH_4_)_2_SO_4_, and 0.05 g/L thiamine-HCl. After autoclaving, the medium was supplemented with 0.5 g/L MgSO_4_·7H_2_O and 3 mL/L of a trace element solution (Table S4). If necessary for plasmid maintenance, the medium was supplemented with 25 µg/mL tetracycline. Precultures of all the *P. putida* strains were initially cultivated in a volume of 5 mL modified Wilms KPi medium supplied with 1% (*w*/*v*) of glucose in conventional glass culture tubes. The cells from these precultures were used for the inoculation of 20 mL modified Wilms KPi medium supplemented with 100 mM acetate for adaptation to the utilization of acetate as the carbon source in 100 mL conventional Erlenmeyer flasks. The cells from the 20-mL cultures adapted to the growth with acetate were subsequently used as the inoculum for the 100-mL main cultures in 500 mL conventional Erlenmeyer flasks in one of the respective medium variants mentioned above. The cultivation of the *P. putida* strains was always carried out at 30 °C while shaking (150× *g*). For the induction of gene expression from the tac promoter within the pVLT31 plasmid, 0.4 mM of isopropyl-β-D-thiogalactopyranoside (IPTG) was added to the medium prior to inoculation.

### 2.2. Bacterial Strains and Plasmids

Aside from the *P. putida* KT2440 strain, two other *P. putida* strains derived from this wild-type strain were constructed and used in this study. For the construction of these strains, the backbone plasmid pVLT31 as well as the plasmid pVLT31_*rhlAB* containing the *rhlAB* genes of the strain *P. aeruginosa* PAO1 were used. All the plasmids used in this study are listed in Table 1. The construction of the pVLT31_*rhlAB* plasmid was carried out by Wittgens et al. (2011) [7]. The aforementioned plasmids were transformed into *E. coli* DH5α and stored in glycerol cryo-stock cultures at −80 °C. To obtain the plasmids, the respective *E. coli* DH5α strains were grown from glycerol stock cultures overnight, and the plasmids were isolated using the QIAprep^®^ Spin Miniprep Kit (Qiagen; Venlo, Netherlands). The plasmid identity was verified via restriction analysis using the enzymes *Sap*I and *Xba*I. To obtain the *P. putida* KT2440 competent, the cells from a 50-mL overnight culture were sedimented a total of four times via centrifugation (3000× *g*, 4 °C, 30 min), being resuspended in different volumes of 300 mM saccharose (50, 25, and 12.5 mL) between each centrifugation step. After the last centrifugation step, the cells were resuspended in a final volume of 750 µL of saccharose, and 250 µL of glycerol (100%) was added. The competent cells were stored at −80 °C in aliquots of 150 µL. The transformation of the pVLT31 and pVLT31_*rhlAB* plasmids into *P. putida* KT2440 was carried out via electroporation. Therefore, the aliquots of the competent *P. putida* cells were thawed on ice, and 500 ng of the desired plasmid DNA was added. The mixtures were transferred to an electroporation cuvette. Electroporation was carried out at 12.5 kV/cm. After that, the cells were mixed with 800 µL of S.O.C. medium (Table S5), transferred into a sterile 15-mL falcon tube, and incubated for 2 h at 30 °C and 150× *g* orbital shaking for cell recovery. The selection of the successfully transformed cells was carried out on LB agar plates containing 25 µg/mL tetracycline. The resulting *P. putida* [pVLT31] and *P. putida* [pVLT31_*rhlAB*] strains were used for the subsequent growth experiments.

In order to avoid possible contaminations of the feeder strain, it was decided to utilize an *A. woodii* strain carrying the empty vector plasmid pMTL83251 with a clarithromycin-resistance gene. The *A. woodii* [pMTL83251] strain was provided by the Institute for Microbiology and Biotechnology of the University of Ulm.

### 2.3. Optical Density (OD_600_) Measurement

For determination of the OD_600_ during the growth experiments, samples of up to 1 mL were obtained from the respective cell cultures. The UV/Vis spectrophotometer UV-1600PC (VWR, Randor, PE, USA) was used to measure the OD_600_. If the resulting absorption values exceeded 0.3, the sample was diluted accordingly with the medium of the type that was used during the respective cultivation.

### 2.4. Quantification of Mono-Rhamnolipids

For the determination of mono-rhamnolipid production, samples of a 100-µL culture suspension were obtained. After centrifugation for 3 min at 14,000× *g*, the supernatants were used for the liquid/liquid extraction of the mono-rhamnolipids. For the rhamnolipid extraction, 500 µL of ethylacetate was added to the culture supernatants and thoroughly mixed. After centrifugation for 30 s at 21,500× *g*, the clear upper phase was obtained. For each sample, the process was repeated three times. In the next step, the solvent was evaporated under the application of a vacuum for 35 min at 60 °C and 1400× *g* using a CentriVap^®^ Benchtop Centrifugal Vacuum Concentrator (Labconco Corporation, Kansas City, MO, USA). The total quantification of the mono-rhamnolipids was conducted with the orcinol assay. The rhamnolipids were resuspended in 100 µL of deionized water. Subsequently, 800 µL of sulfuric acid (60% *v*/*v*) and 100 µL of orcinol solution (1.6% *w*/*v* in deionized water) were added. The samples were then incubated for 30 min at 80 °C and 800× *g* orbital shaking in a thermomixer. After cooling to room temperature, the absorption of the samples at 421 nm was measured in comparison to the extracts of commercial pure mono-rhamnolipids (AGAE Technologies LLC, Corvallis, OR, USA) in different predefined concentrations from 10 mg/L to 1 g/L. If the resulting absorption values exceeded 0.3, the sample was diluted accordingly with a mixture of sulfuric acid and deionized water (4:1).

### 2.5. Gas Chromatography (GC)

The analysis of the acetate concentrations was conducted via gas chromatography (GC) utilizing the Clarus^®^ 600 system (Perkin Elmer, Waltham, MA, USA). The cells present in the samples of all the culture supernatants were pelleted via centrifugation (30 min, 4 °C, 14,000× *g*). A total of 480 µL of supernatant was acidified by adding 20 µL of HCl (2 M). The Clarus^®^ 600 system was equipped with an Elite-FFAP capillary column (Perkin Elmer, Waltham, MA, USA) with a length of 30 m, an inner diameter of 0.32 mm, and a density film of 0.25 µM. A total of 1 µL of the supernatant was injected via the preheated injector (200 °C) with a split ratio of 20 H_2_, which served as the carrier gas, and a flow rate of 45 mL/min. Detection occurred using a flame ionization detector (FID) at a temperature of 300 °C. The analysis of the samples was carried out using the following temperature profile: 2 min at 90 °C, from 90 °C to 250 °C in 40 °C/min, 1 min at 250 °C. External quantification standards were used for the calibration.

### 2.6. Primers and qPCR

During the growth experiments of *P. putida* KT2440 [pVLT31_*rhlAB*] in cell-containing spent medium of *A. woodii*, the investigation of *P. putida* growth and decay of *A. woodii* within the culture was not feasible via measuring the OD_600_. To gain information on the development of the two strains’ respective cell populations, the relative quantification of the selected strain-exclusive genes was carried out via qPCR. In the case of *A. woodii*, the gene *acsA* (Awo_10740), coding for the carbon monoxide dehydrogenase (CODH) catalytic subunit of the bifunctional CO dehydrogenase/acetyl-CoA synthase (ACS) complex was selected as a target. This enzyme plays a central role in CO_2_ fixation via the Wood-Ljungdahl pathway by catalyzing the reduction of one molecule of CO_2_ to CO via the CODH subunit, which is subsequently turned into the carbonyl group of acetyl-CoA via the ACS subunit [43,61,62]. Next, qPCR for the aforementioned gene was carried out using the SK_awo_Fwd1 and SK_awo_Rev1 primers, producing a PCR product of 163 base pairs (bp) in length. For *P. putida*, the gene *lpxD* (PP_RS08260) coding for the UDP-3-*O*-(3-hydroxymyristoyl) glucosamine *N*-acyltransferase enzyme was selected as a genetic target for qPCR. The enzyme plays an important role in the biosynthesis of lipid A and is therefore essential for the formation of lipopolysaccharides (LPSs), which form the external layer of the gram-negative bacterial outer cell membrane [63]. For the qPCR of this gene, the SK_ppu_Fwd1 and SK_ppu_Rev1 primers were used, producing a PCR product with a length of 412 bp. Primer synthesis was conducted using biomers.net GmbH (Ulm, Germany). The sequences of the primers utilized for the PCR processes are provided in Table 2.

In all the PCR processes performed within this study, cycling was performed as follows: 2 min at 95 °C followed by 40 cycles of 15 s at 95 °C, 30 s at 60 °C, and 30 s at 72 °C. The initial verification of the primer performance and specificity was conducted through a conventional PCR process using the isolated genomic DNA (gDNA) of *A. woodii* [pMTL83251] and *P. putida* [pVLT3_*rhlAB*] as templates. To investigate potential unspecific bindings, a PCR was carried out using both primer pairs on each of the bacterial gDNA samples separately. The isolation of bacterial genomic DNA was carried out from 5-mL overnight cultures using the DNeasy^®^ Blood and Tissue Kit (Qiagen, Venlo, Netherlands) according to the handling guidelines provided in the user manual. The resulting PCR products were investigated via electrophoresis in a 1% (*w*/*v*) agarose gel.

For the preparation of the qPCR samples, the PowerTrack^®^ SYBR^®^ Green master mix (Thermo Fisher Scientific; Waltham, MA, USA) was used. For each sample, separate PCR reactions were prepared for each of the two used primer pairs. All reactions were carried out in triplicates. Each single reaction was prepared by adding 5 µL of PowerTrack^®^ SYBR^®^ Green Master Mix (Thermo Fisher Scientific Inc., Darmstadt, Germany), 0.5 µL primer working solution (8 µM) of the desired primer pair, and 3.4 µL of DNase-free water to the respective well. As a template material, 1 µL of the desired bacterial culture suspension was mixed with 0.25 µL of Yellow Sample Buffer (provided within the PowerTrack^®^ master mix) and added into the desired well. The qPCR process was carried out using a qTOWER3^®^ Real-time Thermocycler (Analytik Jena, Jena, Germany). A melting curve (60–95 °C at increments of 0.5 °C) was created for each experiment to verify the specificity of the primer amplification. The investigation of the population development of *A. woodii* [pMTL83251] and *P. putida* [pVLT31_*rhlAB*] was realized by comparing the species-specific C_t_ values of the culture samples to the ones obtained from the measurement of the samples from the pure single cultures of both species with predefined O._600_ values from 0.01 to 2.0. From the measurement values of said single-culture samples, an OD_600_/C_t_ standard curve was created with Origin^®^Pro 2021b software (OriginLab Corporation; Northampton, MA, USA) via a nonlinear regression using the function “ExpDec1” with the following Equation (1):

y = A1 × exp(−x/t1) − y_0_ (1)

This standard curve was subsequently used for the determination of an “OD_600_ equivalence value” to investigate the OD_600_ development in the unknown samples indirectly.

### 2.7. Preservation of the Bacterial Strains

For the preservation of the empty vector strain *P. putida* [pVLT31] and the rhamnolipid producer strain *P. putida* [pVLT31_*rhlAB*], 500 µL of an overnight culture of the respective strain was mixed with a sterile glycerol solution (50% in deionized water (*v*/*v*)), transferred into a sterile cryo-tube, and subsequently stored at −80 °C.

## 3. Results

### 3.1. Cultivation of the P. putida Strains Using Acetate as a Substrate

**Figure 1:**
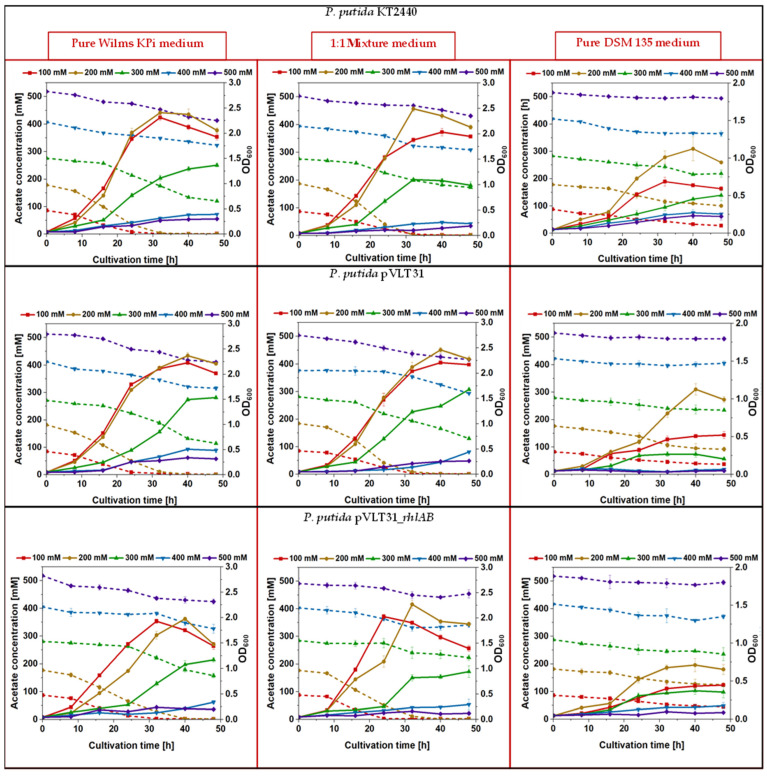
Growth (continuous lines) and acetate consumption (dashed lines) of the wild-type strain *P. putida* KT2440 (first row of the figures), the empty vector strain *P. putida* [pVLT31] (second row of the figures), and the mono-rhamnolipid producing strain *P. putida* [pVLT31_*rhlAB*] (third row of the figures). For each strain, cultivation was carried out in modified Wilms KPi medium (first column of the figures) in a 1:1 mixture of modified Wilms KPi medium and modified DSM 135 medium (second column of the figures) and in pure modified DSM 135 medium (third column of the figures). For each combination, cultivation was carried out separately with 100, 200, 300, 400, and 500 mM acetate as the carbon source. The error bars represent the deviations of the biological triplicates.

**Figure 2:**
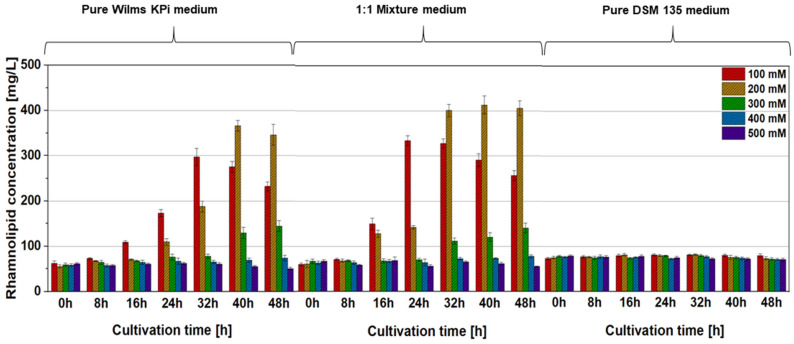
Production of mono-rhamnolipids by *P. putida* [pVLT31_*rhlAB*] supplemented with 100 mM (red), 200 mM (brown), 300 mM (green), 400 mM (blue), or 500 mM (purple) acetate as the carbon source, respectively. Cultivation was carried out in modified Wilms KPi medium in a 1:1 mixture of modified Wilms KPi medium and modified DSM 135 medium and in pure modified DSM 135 medium. The error bars represent the standard deviation of the biological triplicates.

**Figure 3:**
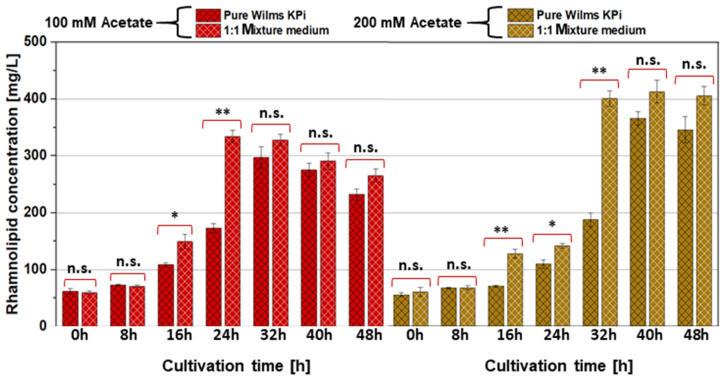
Comparison of the mono-rhamnolipid production of *P. putida* [pVLT31_*rhlAB*] supplemented with 100 mM (red) and 200 mM (brown) acetate as the carbon source cultivated in pure modified Wilms KPi medium (black grid columns) and in a 1:1 mixture medium of modified Wilms KPi medium and modified DSM 135 medium (white grid columns). The statistical significance of the differences of the rhamnolipid concentrations reached with pure Wilms KPi medium and 1:1 mixture medium is illustrated. The error bars represent the deviations of the biological triplicates. *: *p*-value > 0.05; ** *p*-value > 0.01: n.s.: not significant.

Our concept was based on the use of *A. woodii* as a “feeder strain” and recombinant *P. putida* as the “producer strain” for the recombinant product of rhamnolipids. Both bacterial strains have well-established growth media, which, however, differ considerably in their composition [39,50,51,54,58]. The intention was to use acetate from *A. woodii* cell cultures as the source for the growth of *P. putida* and the production of rhamnolipids in the form of spent medium. Thus, in the first step, the growth behavior of the wild-type strain *P. putida* KT2440 and the recombinant strains *P. putida* [pVLT31] and *P. putida* [pVLT31_*rhlAB*] was investigated in modified Wilms KPi (the dedicated *P. putida* medium), modified DSM 135 (the dedicated *A. woodii* medium), and a 1:1 mixture of both, supplemented with different concentrations of acetate as the carbon source (100, 200, 300, 400, and 500 mM, respectively). Interestingly, the growth of *P. putida* was drastically reduced in pure, modified DSM 135 medium, with cell densities reaching not more than 50% compared to modified Wilms KPi (Figure 1). Up to a concentration of 200 mM of acetate, the consumption of acetate was complete within the first 30 h of cultivation in both the pure modified Wilms KPi medium and the 1:1 mixture medium, with comparable cell densities for the respective strains. Higher acetate concentrations appeared to inhibit *P. putida* growth in all the media (Figure 1). The cell densities of *P. putida* harboring the pVLT31_*rhlAB* plasmid for the recombinant production of rhamnolipids were, as expected, slightly lower compared to the wild-type and the empty-vector control strain. However, the 1:1 mixture medium had no negative effect on the growth of the cells (Figure 1). The highest rhamnolipid concentration of approximately 400 mg/L was reached within the 1:1 mixture medium after 40 h of cultivation, which is a considerable dimension for this strain in a shake flask experiment without prior optimization (Figure 2) [32,36]. The observed final rhamnolipid concentrations reached within the modified Wilms KPi medium, and the 1:1 mixture medium supplied with either 100 mM or 200 mM of acetate showed no significant difference, indicating that the latter medium also had no negative effect on the recombinant production (Figure 3).

### 3.2. Production of Mono-Rhamnolipids by P. putida [pVLT31_rhlAB] in Cell-Free A. woodii [pMTL83251] Spent Medium

**Figure 4:**
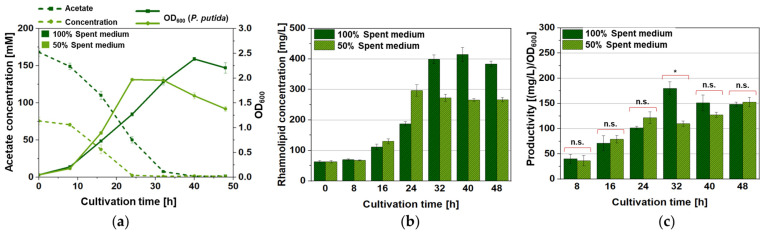
Growth (continuous lines) and acetate consumption (dashed lines), (**a**) mono-rhamnolipid-production (**b**) and productivity values with the statistical analyses of the productivity differences (**c**) of *P. putida* [pVLT31_*rhlAB*] grown in different shares of cell-free *A. woodii* [pMTL83251] spent medium. Cultivation was either carried out in pure cell-free spent medium (dark green) obtained from the Awo2 culture (Figure S1) or in a 1:1 mixture of spent medium obtained from the Awo1 culture (Figure S1) and fresh modified Wilms KPi medium (light green) without additional acetate. The error bars represent the standard deviations of the technical triplicates, *: *p*-value > 0.05; n.s.: not significant.

The production of acetate in the “feeding cultures” of *A. woodii* [pMTL83251] with CO_2_ + H_2_ was carried out in a modified DSM 135 medium for 120 h and yielded a final concentration of approximately 150 mM and an OD_600_ of the cell cultures of typically 1.5–1.7, as expected (Figure S1) [47,50]. After *A. woodii* cell removal, both the pure spent medium and the spent medium diluted 1:1 with fresh Wilms KPi medium supported the growth of the *P. putida* [pVLT31_*rhlAB*] rhamnolipid producer strain (Figure 4a), reaching optical densities of the cell cultures of approximately 2.0 for the 1:1 mixture medium and approximately 2.5 for the pure spent medium containing 75 mM and 150 mM acetate for the 1:1 mixture medium and the pure spent medium, respectively. The acetate concentration after the dilution thus resulted in cell densities comparable to those obtained in the reference experiments with pure acetate as the carbon source in the respective basic media (compare with Figure 1). The same is true for the rhamnolipid production, delivering final concentrations between 300 and 400 mg/L, which are in the same range as in the former experiments (compare with Figure 2). Interestingly, in contrast to the initial experiments where pure DSM 135 medium was less suited for *P. putida* growth, the pure DSM 135-based spent medium appeared to result in a considerable surplus of cell density and rhamnolipid titer after cultivation for 32, 40, and 48 h (Figure 4b,c). The values of rhamnolipid productivity (mg/L per OD_600_) generally showed no significant difference between the pure modified DSM-based spent medium cultures and the ones prepared within the 1:1 mixture medium, indicating that dilution with fresh modified Wilms KPi medium does not benefit recombinant rhamnolipid production and can be omitted in this process (Figure 4c).

### 3.3. Production of Mono-Rhamnolipids by P. putida [pVLT31_rhlAB] in Cell-Containing A. woodii [pMTL83251] Spent Medium (Culture Broth)

**Figure 5:**
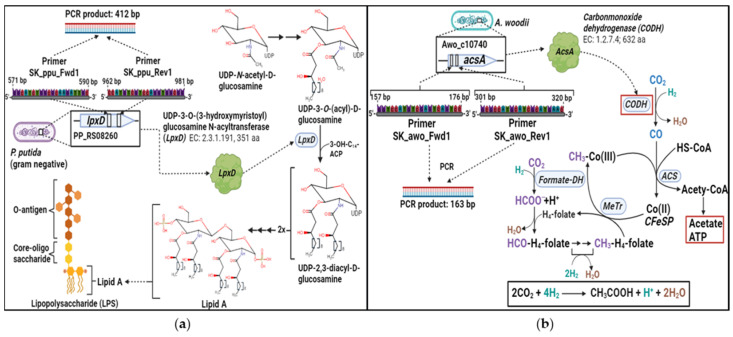
Illustration of species-exclusive target genes selected for qPCR. For the detection of the gram-negative bacterium *P. putida*, the gene *lpxD* (PP_RS08260) was chosen as the target. The gene encodes UDP-3-*O*-(3-hydroxymyristoyl) glucosamine *N*-acyltransferase, which plays an important role in the biosynthesis of lipid A, a central component of lipopolysaccharides (LPSs) commonly found forming the outermost layer of a gram-negative cell wall. (**a**) The target gene chosen for the detection of *A. woodii* was *acsA* (Awo_c10740), which encodes the carbon monoxide dehydrogenase (CODH) subunit of the bifunctional CODH/acetyl-CoA synthase (ACS) enzyme complex. This subunit is responsible for the introduction of one of the two CO_2_ molecules into the Wood-Ljungdahl pathway (**b**).

The next aim of our proof-of-concept-study presented here was to evaluate whether it was possible to avoid cell removal from the feeder cultures prior to the cultivation of the producer strain for possible future processes, which would represent a considerable simplification of the procedure and in turn an increase in the economical perspective of such sequential production processes [64]. Keeping *A. woodii* cells in the spent medium, however, poses an experimental difficulty, as the growth of *P. putida* in such cultures cannot be easily monitored by measuring the optical density. Thus, we decided to instead measure the relative amount of marker genes in the chromosomes of both *P. putida* and *A. woodii* referenced by the standard curves of the defined optical density values. *P. putida* KT2440 is a gram-negative bacterium, therefore it appeared reasonable to choose a gene encoding for a central enzyme of the lipid A biosynthesis as the marker gene, since lipid A is the essential component of lipopolysaccharides in the cell wall of gram-negative bacteria. In contrast, *A. woodii* is a gram-positive bacterium, thus not containing LPSs. Moreover, *A. woodii* harbors the Wood-Ljundahl pathway, leading us to choose one of its enzyme genes as the marker gene, allowing us to specifically distinguish *A. woodii* from *P. putida* KT2440. Primer pairs were designed resulting in sizes of the intended PCR products of 412 bp for *P. putida* KT2440 (Figure 5a) and 163 bp for *A. woodii* (Figure 5b). The specificity of the primer sets was verified after PCR in a traditional agarose gel (Figure S2). Next, standard curves were obtained from serial dilutions of cultures in triplicates, representing optical densities ranging from 0.01 to 2.0 for both strains (Figures S3 and S4). This monitoring principle was tested in a series of experiments with artificial mixtures of both cultures in different ratios from 15:1 to 1:1 (*P. putida* to *A. woodii*) and vice versa. Despite relatively high deviations from the calculated to predefined optical densities of up to 33% for a given OD_600_ of *P. putida* of 0.3 and a measured/calculated OD_600_ of 0.2 (i.e., in the range of extremely low cell densities), the measurements were precise enough to reflect the respective predefined amounts of cells and to allow the monitoring of the relative content of both strains along a growth curve of *P. putida* and a “decay curve” of *A. woodii* in the presence of *P. putida* (Figure S5, Table S6).

**Figure 6:**
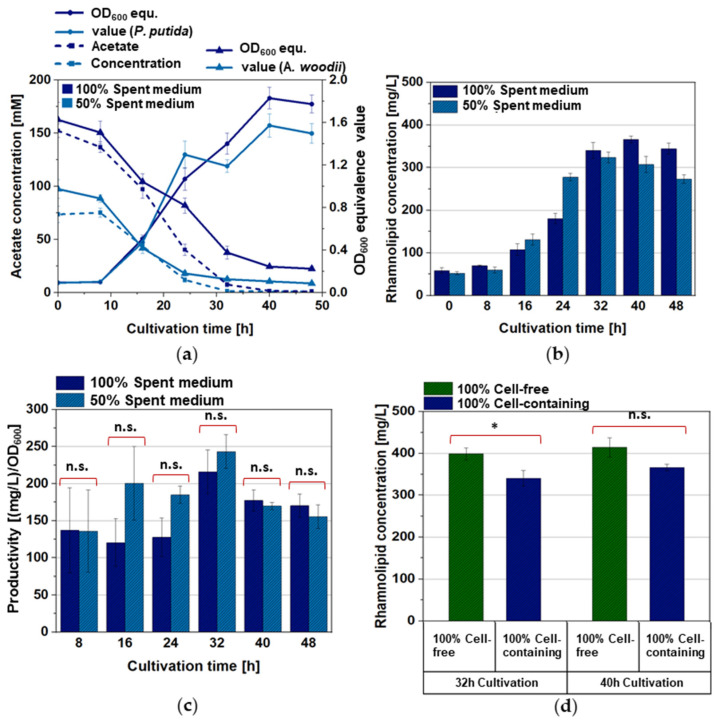
Growth of *P. putida* [pVLT31_*rhlAB*], decay of *A. woodii* [pMTL83251], and acetate consumption (dashed lines) (**a**) as well as mono-rhamnolipid concentrations (**b**) and productivity values in concentration per OD_600_, with the statistical significance of the productivity differences (**c**) in the different shares of cell-containing spent medium of *A. woodii* [pMTL83251]. The OD_600_ equivalence (equ.) values were calculated from the data of qPCR for the genes exclusive to the respective bacterial species that were compared to the ones obtained from a standard curve. Cultivation was either carried out in pure cell-containing spent medium (dark blue) obtained from the Awo4 culture (Figure S1) or in a 1:1 mixture of spent medium obtained from the Awo3 culture (Figure S1) and fresh modified Wilms KPi medium (blue) without additional acetate. Further illustrated is a comparison of the overall highest reached concentrations of mono-rhamnolipids, obtained in pure cell-free and cell-containing *A. woodii* spent medium, with the statistical significance of the concentration differences. (**d**) The error bars represent the standard deviations of the technical triplicates, *: *p*-value > 0.05; n.s.: not significant.

The following key experiment proved the idea that the growth of *P. putida* and the production of rhamnolipids can be achieved using raw (cell-containing) culture broth of *A. woodii* cultures; rhamnolipid concentrations of 340 mg/L after 32 h and 366 mg/L after 40 h were obtained compared to 399 mg/L and 414 mg/L, respectively, after 32 and 40 h of cultivation in the cell-free spent medium (Figure 4b and Figure 6b). The number of *A. woodii* cells measured via qPCR decreased, indicating a disintegration of the cell structure exposing the chromosome to degradation (Figure 6a). Similar to the cultivations conducted in the cell-free spent medium, the productivity was not significantly different for the cultivations in the 1:1 mixture medium and pure cell-containing spent medium (culture broth) (Figure 6c), suggesting again that the dilution step with fresh modified Wilms KPi medium could be circumvented without a loss in the efficiency of recombinant rhamnolipid production. The comparison of the highest final concentrations reached after 32 and 40 h revealed a significant but small difference only after 32 h between the cell-free and cell-containing *A. woodii* spent medium (Figure 6d). Thus, careful consideration of the advantage of a slightly higher final concentration in cell-free spent medium and the necessity for additional technical efforts for cell separation appears to be reasonable for potential future implementation in real processes.

## 4. Discussion

Recently, we introduced acetate as an attractive new and alternative substrate for the recombinant production of rhamnolipids in *P. putida* KT2440 [40]. In particular, acetate originating from fermentations based on renewable carbon sources, such as anaerobic gas fermentation, has been discussed as probably being the ideal alternative to allow the establishment of sustainable production processes for various biocommodities [45,53,64].

The aim of this project was to demonstrate the possible utilization of acetate-containing spent medium obtained from *A. woodii* anaerobic gas fermentation as the nutrient source for the growth of *P. putida* KT2440 and the production of rhamnolipids via a sequential fermentation approach. Although our initial experiments suggested a notably reduced growth of *P. putida* in the dedicated *A. woodii* medium DSM 135 supplemented with acetate compared to the modified Wilms KPi medium as the usual *P. putida* medium, this drawback did not occur in the 1:1 mixture of both media. As described by Hoffmeister et al. (2016) [50], the modified DSM 135 medium was supplemented with a selenite-tungstate solution, which is known to exhibit a beneficial effect on the autotrophic growth of *A. woodii*. Tungsten is already known to be present in the formate dehydrogenase of other acetogens, such as *Moorella thermoacetica* [65,66], which is a central enzyme of the Wood-Ljungdahl pathway in all acetogenic bacteria. It has, however, been shown to have toxic effects on different species of *Pseudomonas* [67,68,69]. The same medium, as the spent medium variant after cultivation of *A. woodii*, however, worked perfectly to cultivate *P. putida,* suggesting that the complexation of the heavy metal tungsten in cellular components of the feeder strain removed it from the accessible medium, thereby potentially omitting toxicity toward growing *Pseudomonas* cells. In light of this fact, our initial reasoning to dilute the spent media with fresh dedicated *Pseudomonas* medium became, in principle, obsolete. Rhamnolipid production using the recombinant *P. putida* KT2440 [pVLT31_*rhlAB*] was possible independent from whether spent medium with or without *A. woodii* cells was used and concentrations as well as productivities obtained were in a similar range. The total amounts of rhamnolipids of 366–414 mg/L produced after 40 h of cultivation were in the same range of rhamnolipid concentrations observed in previous studies in also unoptimized shake flask experiments with lignocellulose-derived sugars and acetate derived from pyrolysis oil [40].

The concept to use spent media still containing the biomass of the feeder strain *A. woodii* is accompanied by the difficulty to determine the correct cell densities using simple optical measurements. The qPCR strategy presented here, which was based on the use of primer pairs directed against the genes encoding for essential and exclusive metabolic functions of both *A. woodii* [pMTL83251] and *P. putida* [pVLT31_*rhlAB*], turned out to be suitable for a relatively precise estimation of the content of both cell types simultaneously in the rhamnolipid production cultures. The abundance of *A. woodii* DNA in the cultures was reduced with cultivation time, suggesting that the cell integrity was compromised and thus the DNA was exposed to degradation. This, however, does not necessarily indicate that *A. woodii* biomass is consumed in the process as an additional source of nutrients by *P. putida* for the growth and recombinant production of rhamnolipids. In-depth studies of the consumption of further *A. woodii* cell components, such as proteins, polysaccharides, and lipids, are urgently required to describe and understand the role of *A. woodii* biomass in this process.

In summary, our initial experiments presented here represent an important proof of concept that the production of rhamnolipids based on acetate/biomass from anaerobic gas fermentation of the *A. woodii* feeder strain is possible and reaches reasonable productivities and final concentrations. Nevertheless, there is an enormous space for optimization by bioengineers to set up a real biotechnological process. We thus have added recombinant rhamnolipids to the constantly growing portfolio of value-added commodity chemicals either directly or indirectly produced in cultivations based on gas-fermenting acetogens. We conclude that sequential fermentation processes of this type represent a yet inconceivably high potential for future truly sustainable bioprocesses and will open new avenues towards a successful transformation of human technology into the direction of a decarbonized global industry.

## Figures and Tables

**Table 1:** Plasmids used in this study and descriptions of their relevant features.

Plasmid	Description	Source
pVLT31	Expression vector with the IPTG-inducible tac promoter, TetR	De Lorenzo et al., (1993) [59]
pVLT31_*rhlAB*	Same as pVLT31, *rhlAB* genes of *P. aeruginosa* PAO1 under the control of the tac promoter	Wittgens et al., (2011) [7]
pMTL83251	The backbone for homologous recombination vectors, CmR	Heap et al., (2009) [60]

**Table 2:** Primers used for relative gene quantification via qPCR.

Primer	Sequence (5′ → 3′)
SK_awo_Fwd1	GGTCCATGTCGAGTCAGTCC
SK_awo_Rev1	GCGCCATGCATCGCATGAAC
SK_ppu_Fwd1	GGCATCTGGCGCAAGATTGC
SK_ppu_Rev1	CCATGTCGTCCAGGTGGCGG

## Data Availability

The data can be requested from the authors for valid reasons.

## Supplementary Materials

The following supporting information can be downloaded at: https://www.mdpi.com/article/10.3390/microorganisms12030529/s1, Figure S1: Growth (a) and final acetate concentrations (b) of four cultures of *A. woodii* [pMTL83251] (Awo1–4) after 120 h of cultivation. The red horizontal line represents the average final acetate concentration (153.84 mM) of the four cultures. Figure S2: Visualization of the PCR products obtained using the primer pairs SK_awo_Fwd1+Rev1 (163 bp) and SK_ppu_Fwd1+Rev1 (412 bp) respectively in a 1 % (*w*/*v*) agarose gel. To investigate the primers specificity, genomic DNA of both *A. woodii* and *P. putida* were used as template material for each of the primer pairs separately. To detect potential contaminations of the used diluent with foreign genomic material, PCR for both primer pairs was additionally carried out with PCR water. *NTC: Non-template control. Figure S3: Results of qPCR with culture suspension of *P. putida* [pVLT31_*rhlAB*] with different OD_600_ values from 0.01 to 2.0 used as template material. The shown data were obtained using the primer pair SK_ppu_Fwd1+Rev1 for specific detection of the *lpxD* (PP_08260) gene of *P. putida* KT2440. Curves of matching colour represent technical triplicates (a). The mean C_t_ values obtained from qPCR were plotted against the corresponding OD_600_ values to obtain a standard curve via nonlinear regression, using a function corresponding to Equation (1) (b). Figure S4: Results of qPCR with culture suspension of *A. woodii* [pMTL83251] with different OD_600_ values from 0.01 to 2.0 used as template material. The shown data were obtained using the primer pair SK_awo_Fwd1+Rev1 for specific detection of the *acsA* (Awo_c10740) gene of *A. woodii*. Curves of matching colour represent technical triplicates (a). The mean C_t_ values obtained from qPCR were plotted against the corresponding OD_600_ values to obtain a standard curve via nonlinear regression, using a function corresponding to Equation (1) (b). Figure S5: Results of qPCR with mixed cell suspensions of *A. woodii* [pMTL83251] and *P. putida* [pVLT31_*rhlAB*] of different predefined OD_600_ ratios used as template material (see Table S6). The values were obtained using the primer pairs SK_awo_Fwd1+Rev1 (a) and SK_ppu_Fwd1+Rev1 (b), respectively. Curves of matching colour represent technical triplicates. Table S1: Composition of SL-9 trace element solution (Tschech and Pfennig, 1984) [52] for preparation of the modified DSM 135 medium. Table S2: Composition of vitamin solution (Wolin et al., 1963, modified) [53] for preparation of the modified DSM 135 medium. Table S3: Composition of selenite-tungstate solution (Tschech and Pfennig, 1984) [53] for preparation of the modified DSM 135 medium. Table S4: Composition of the trace element solution (Wilms et al., 2001) [54] for preparation of modified Wilms KPi medium. Table S5: Composition of S.O.C. medium used for cell regeneration of *P. putida* KT2440 after transformation with plasmids pVLT31 and pVLT31_*rhlAB*. Table S6: Results of OD_600_ calculation from C_t_ values obtained from qPCR with predefined *A.woodii*/*P. putida* mixtures of different OD_600_ ratios used as template material. Also shown are the respective percental deviations of the calculated OD_600_ values from the actual ones. Values of standard deviation refer to technical triplicates.
