# Supplementary material for: Recombinant Production of Pseudomonas aeruginosa Rhamnolipids in P. putida KT2440 on Acetobacterium woodii Cultures Grown Chemo-Autotrophically with Carbon Dioxide and Hydrogen
Source: Microorganisms. 2024 Mar 6;12(3):529. doi: 10.3390/microorganisms12030529 (PMC10972445; doi:10.3390/microorganisms12030529)
Supplement: Supplementary file 1 [file microorganisms-12-00529-s001.zip › microorganisms-2884150-supplementary.pdf]

**Supplementary material**

**Table S1.** Composition of SL-9 trace element solution (Tschech and Pfennig, 1984), [52] for preparation of the modified DSM 135 medium.

| Compound | Concentration [g/L] |
|---|---|
| Nitrilotriacetic acid | 12.8 |
| $MnCl_2$ x 2 $H_2O$ | 0.1 |
| $FeCl_2$ x 4 $H_2O$ | 2 |
| $CoCl_2$ x 6 $H_2O$ | 0.2 |
| $ZnCl_2$ | 0.07 |
| $CuCl_2$ x 6 $H_2O$ | 0.002 |
| $H_3BO_3$ | 0.006 |
| $Na_2MoO_4$ x 2 $H_2O$ | 0.036 |
| $NiCl_2$ x 6 $H_2O$ | 0.024 |

**Table S2.** Composition of vitamin solution (Wolin *et al*., 1963, modified), [53] for preparation of the modified DSM 135 medium.

| Compound | Concentration [g/L] |
|---|---|
| D-biotin | 0.025 |
| Folic acid | 0.025 |
| Pyridoxine-HCl | 0.05 |
| Thiamine-HCl x 2 $H_2O$ | 0.05 |
| Riboflavin | 0.05 |
| Nicotinic acid | 0.05 |
| D-Ca-pantothenate | 0.05 |
| Cyanocobalamine | 0.025 |
| $\alpha$-Aminobenzoic acid | 0.05 |
| $\alpha$-Lipoic acid | 0.025 |

**Table S3.** Composition of selenite-tungstate solution (Tschech and Pfennig, 1984), [53] for preparation of the modified DSM 135 medium.

| Compound | Concentration [g/L] |
|---|---|
| NaOH | 0.5 |
| $Na_2SeO_3$ x 5 $H_2O$ | 0.03 |
| $Na_2WO_4$ x 2 $H_2O$ | 0.04 |

**Table S4.** Composition of the trace element solution (Wilms *et al.*, 2001), [54] for preparation of modified Wilms KPi medium.

| Compound | Concentration [g/L] |
|---|---|
| $ZnSO_4$ x 7 $H_2O$ | 0.18 |
| $CuSO_4$ x 5 $H_2O$ | 0.16 |
| $MnSO_4$ x $H_2O$ | 0.1 |
| $FeCl_3$ x 6 $H_2O$ | 13.92 |
| EDTA | 10.05 |
| $CoCl_2$ x 6 $H_2O$ | 0.18 |
| $CaCl_2$ x 2 $H_2O$ | 0.662 |
| Thiamine-HCl | 10 |

**Table S5.** Composition of S.O.C. medium used for cell regeneration of *P. putida* KT2440 after transformation with plasmids pVLT31 and pVLT31_*rhlAB*.

| Compound | Concentration |
|---|---|
| Tryptone | 2 % (w/v) |
| Yeast extract | 0.5 % (w/v) |
| NaCl | 10 mM |
| KCl | 2.5 mM |
| $MgCl_2$ | 10 mM |
| $MgSO_4$ x7 $H_2O$ | 10 mM |
| $CaCl_2$ x 2 $H_2O$ | 0.662 mM |
| Glucose[1] | 20 mM |

[1]Was added after autoclaving.

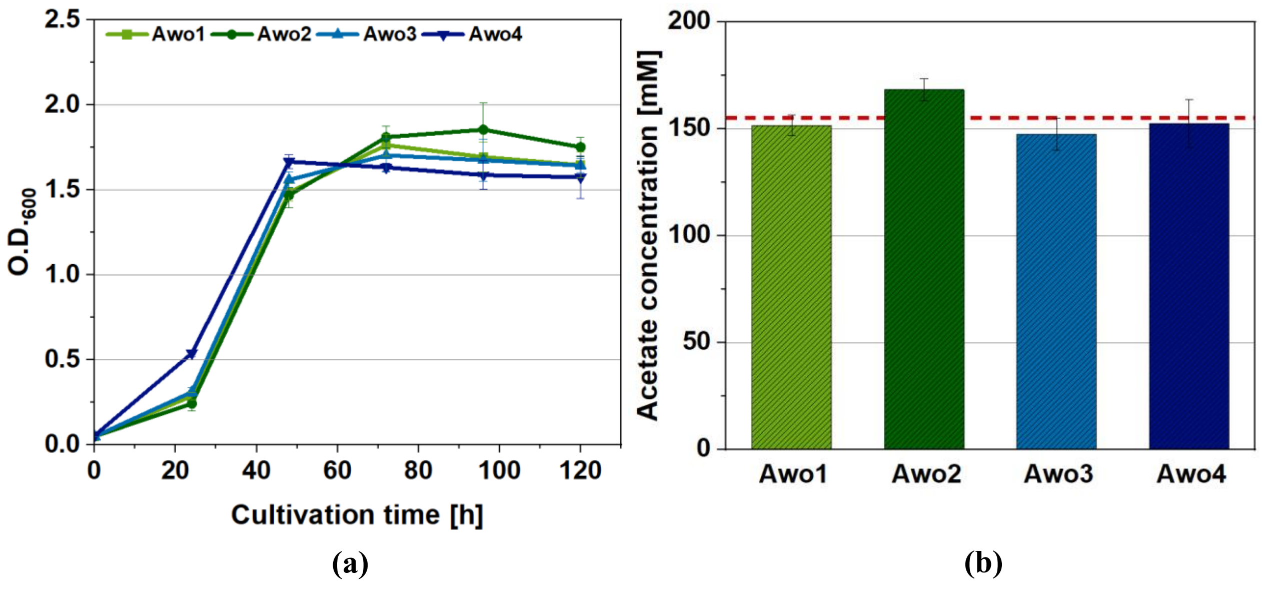


**Figure S1.** Growth **(a)** and final acetate concentrations **(b)** of four cultures of *A. woodii* [pMTL83251] (**Awo1-4**) after 120 hours of cultivation. The red horizontal line represents the average final acetate concentration (153.84 mM) of the four cultures.

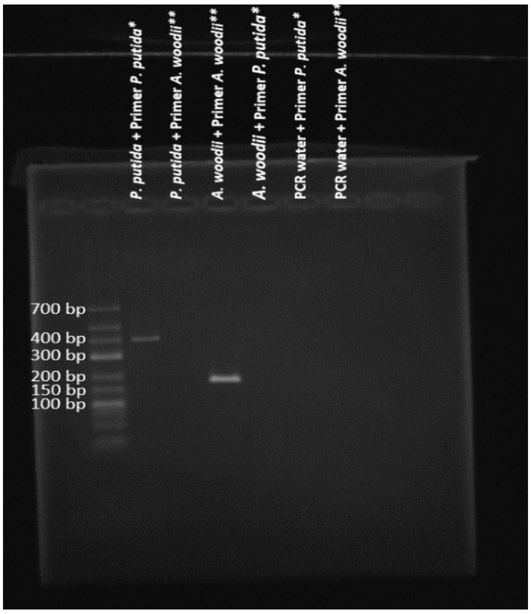


*Refers to the primer pair SK_ppu_Fwd1+Rev1.
**Refers to the primer pair SK_awo_Fwd1+Rev1.

**Figure S2.** Visualization of the PCR products obtained using the primer pairs SK_awo_Fwd1+Rev1 (163 bp) and SK_ppu_Fwd1+Rev1 (412 bp) respectively in a 1 % (w/v) agarose gel. To investigate the primers specificity, genomic DNA of both *A. woodii* and *P. putida* were used as template material for each of the primer pairs separately. To detect potential contaminations of the used diluent with foreign genomic material, PCR for both primer pairs was additionally carried out with PCR water. *NTC: Non-template control.

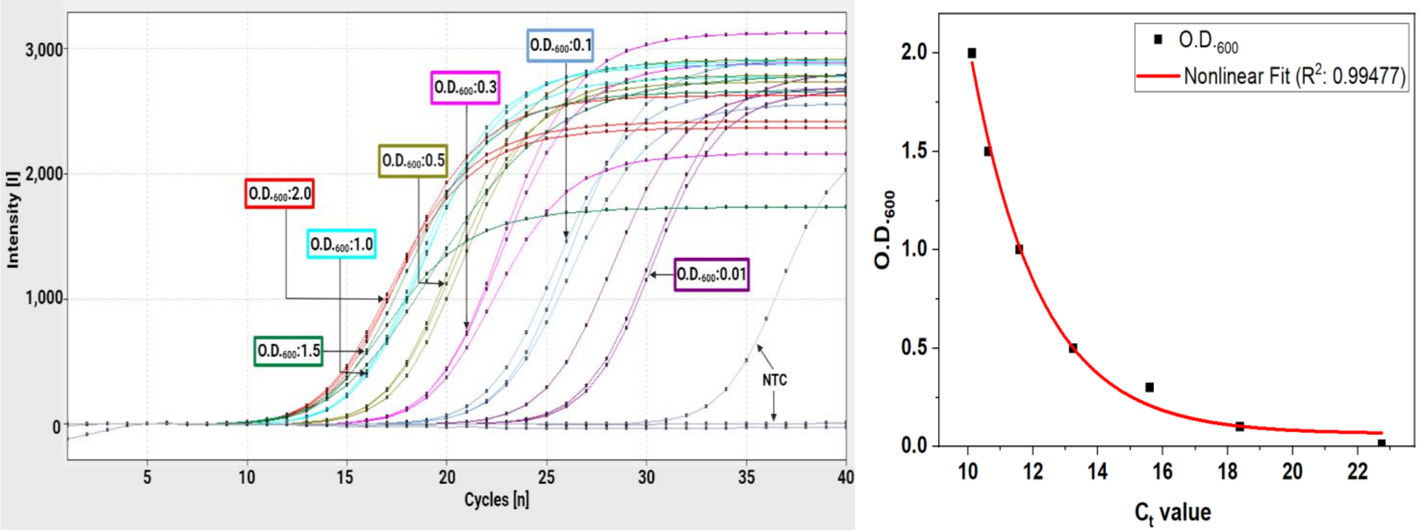


**(a)** **(b)**

**Figure S3.** Results of qPCR with culture suspension of *P. putida* [pVLT31_*rhlAB*] with different $OD_{600}$ values from 0.01 to 2.0 used as template material. The shown data were obtained using the primer pair SK_ppu_Fwd1+Rev1 for specific detection of the *lpxD* (PP_08260) gene of *P. putida* KT2440. Curves of matching colour represent technical triplicates **(a)**. The mean $C_t$ values obtained from qPCR were plotted against the corresponding $OD_{600}$ values to obtain a standard curve via nonlinear regression, using a function corresponding to **Equation 1 (b)**.

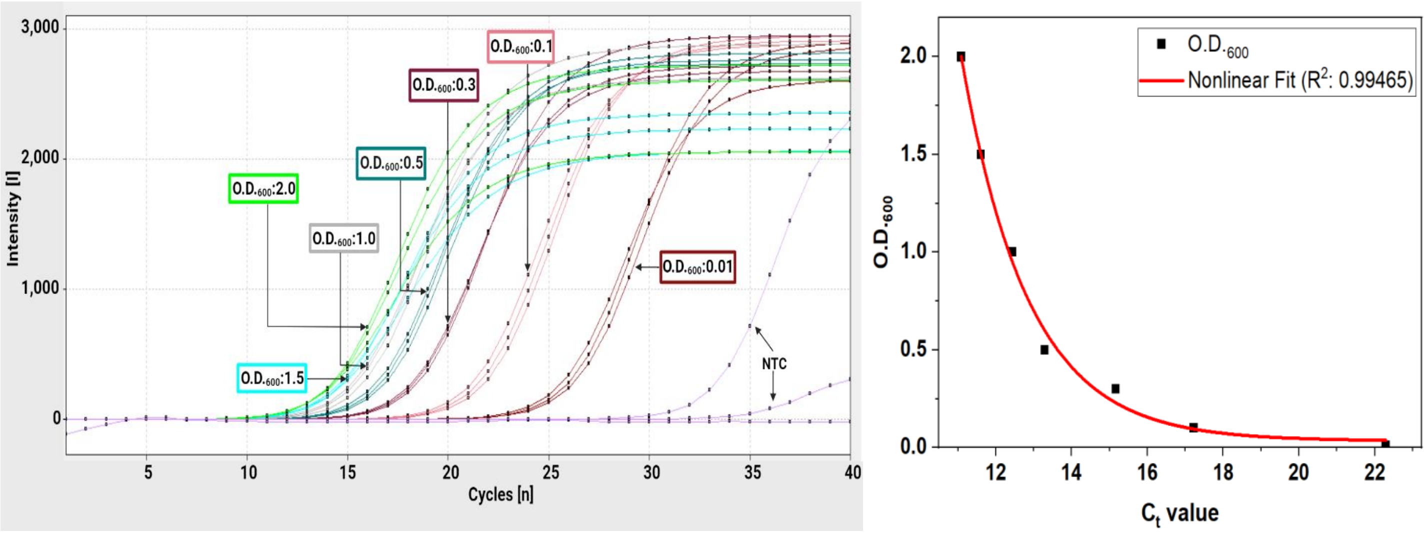


**(a)** **(b)**

**Figure S4.** Results of qPCR with culture suspension of *A. woodii* [pMTL83251] with different $OD_{600}$ values from 0.01 to 2.0 used as template material. The shown data were obtained using the primer pair SK_awo_Fwd1+Rev1 for specific detection of the *acsA* (Awo_c10740) gene of *A. woodii*. Curves of matching colour represent technical triplicates **(a)**. The mean $C_t$ values obtained from qPCR were plotted against the corresponding $OD_{600}$ values to obtain a standard curve via nonlinear regression, using a function corresponding to **Equation 1 (b)**.

**Table S6.** Results of $OD_{600}$ calculation from $C_t$ values obtained from qPCR with predefined *A.woodii*/*P. putida* mixtures of different $OD_{600}$ ratios used as template material. Also shown are the respective percental deviations of the calculated $OD_{600}$ values from the actual ones. Values of standard deviation refer to technical triplicates.

| **Mix-ratio ($OD_{600}$*A. woodii*/$OD_{600}$ *P. putida*)** | ***A. woodii* $OD_{600}$** | ***P. putida* $OD_{600}$** | ***A. woodii* $OD_{600}$ calculated through $C_t$ values** | ***P. putida* $OD_{600}$ calculated through $C_t$ values** | **% deviation $OD_{600}$ (*A. woodii*)** | **% deviation $OD_{600}$ (*P. putida*)** |
|---|---|---|---|---|---|---|
| 15:1 | 1.5 | 0.1 | 1.3 ± 0.061 | 0.1 ± 0.005 | -13.34 | 0 |
| 10:3 | 1.0 | 0.3 | 1.1 ± 0.119 | 0.2 ± 0.017 | +10 | -33 |
| 1:1 | 0.5 | 0.5 | 0.5 ± 0.013 | 0.4 ± 0.019 | 0 | -20 |
| 3:10 | 0.3 | 1.0 | 0.2 ± 0.009 | 0.9 ± 0.107 | -33 | -10 |
| 1:15 | 0.1 | 1.5 | 0.1 ± 0.003 | 1.2 ± 0.111 | 0 | -20 |

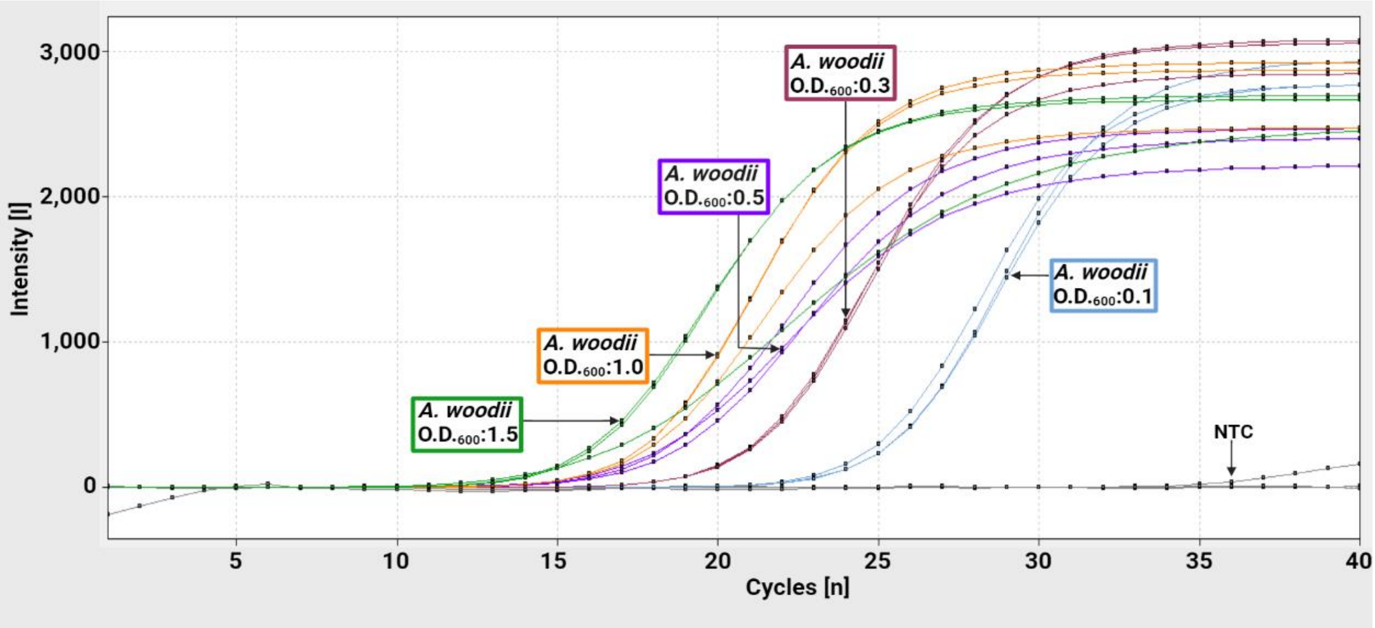


**(a)**

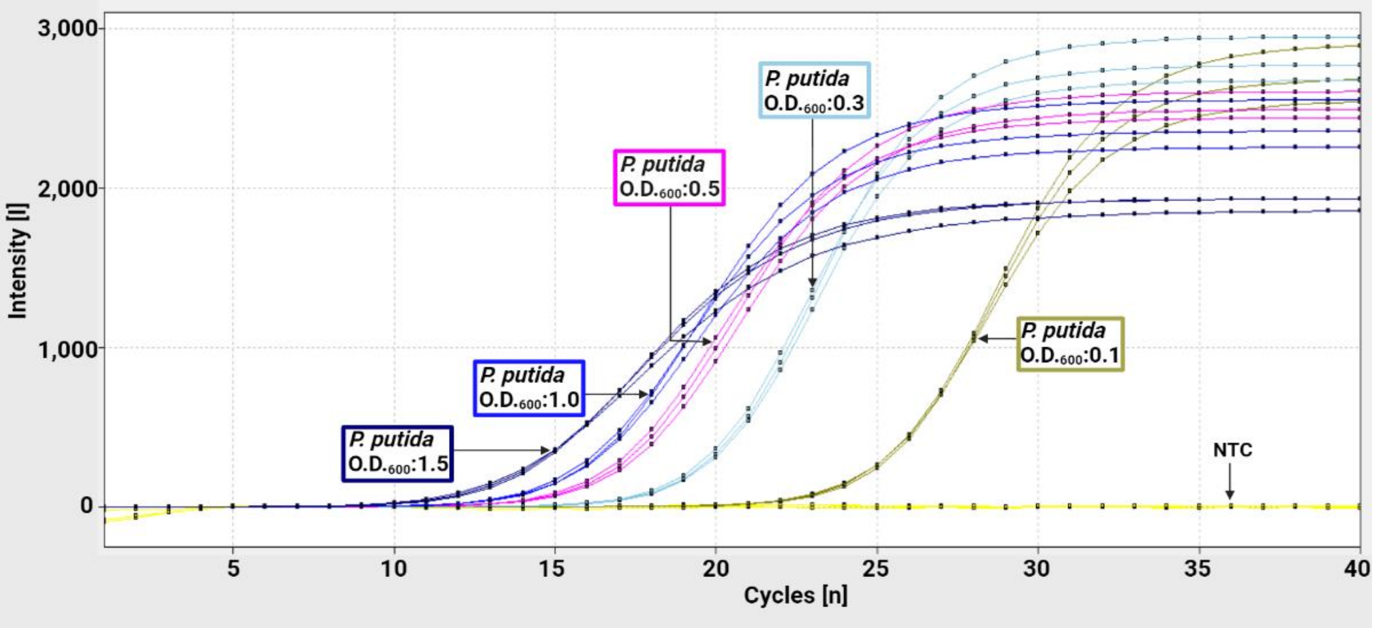


**(b)**

**Figure S5.** Results of qPCR with mixed cell suspensions of *A. woodii* [pMTL83251] and *P. putida* [pVLT31_*rhlAB*] of different predefined $OD_{600}$ ratios used as template material (see **Table S6**). The values were obtained using the primer pairs SK_awo_Fwd1+Rev1 **(a)** and SK_ppu_Fwd1+Rev1 **(b)**, respectively. Curves of matching colour represent technical triplicates.
